# Early childhood education language environments: considerations for research and practice

**DOI:** 10.3389/fpsyg.2023.1202819

**Published:** 2023-09-22

**Authors:** Jennifer Finders, Ella Wilson, Robert Duncan

**Affiliations:** ^1^Department of Human Development and Family Science, Purdue University, West Lafayette, IN, United States; ^2^Department of Speech, Language, and Hearing Sciences, Purdue University, West Lafayette, IN, United States

**Keywords:** language development, early childhood education, assessments, instructional practices, quality

## Abstract

The importance of developing early language and literacy skills is acknowledged by the United Nations Educational, Scientific, and Cultural Organization (UNESCO) as a global human rights issue. Indeed, research suggests that language abilities are foundational for a host of cognitive, behavioral, and social–emotional outcomes. Therefore, it is critical to provide experiences that foster language acquisition across early learning settings. Central to these efforts is incorporating assessments of language environments into research and practice to drive quality improvement. Yet, several barriers may be preventing language environment assessments from becoming widely integrated into early education. In this brief, we review evidence on the types of experiences that promote language development, describe characteristics of language environment assessments, and outline practical and philosophical considerations to assist with decision-making. Further, we offer recommendations for future research that may contribute knowledge regarding strategies to assess and support language development. In addressing both areas, we highlight the potential for early childhood language environments to advance equity.

## Introduction

Despite compelling evidence indicating the importance of environmental inputs for language acquisition within the first 5 years of life (e.g., Anderson et al., 2021), several obstacles have prevented language environment assessments from becoming widely integrated into early education settings (e.g., Mitchell, 2016). First, for any program that seeks to enhance language environments, measuring the processes known to drive language development is fundamental prior to developing intervention strategies and monitoring continual quality improvement. Yet, the limited application of language environment assessments in interventions or programs of scale restricts our understanding of how to effectively utilize language assessments to evaluate practice (e.g., Greenwood et al., 2020). Thus, we offer opportunities for future research that have the potential to contribute knowledge regarding strategies to assess and support language development and fill critical gaps in the literature. Second, selecting a language environment assessment that meets program goals, can be administered with fidelity, is accommodating to resource constraints, and is appropriate for informing professional development can be arduous (e.g., Zaslow et al., 2009; Derrick-Mills et al., 2014; Ackerman, 2019). To help practitioners overcome this challenge, we review evidence on the types of experiences that promote language development, describe characteristics of language environment assessments, and outline practical and philosophical considerations to assist with decision-making. The overall goal of this mini review is to demystify the complexities of early childhood language environments and articulate the ways in which language environment assessments can drive practice. We believe this is an essential step toward improving early language experiences and evaluating the impacts of education programming on learning in this fundamental developmental domain.

## Environments that support early language development

Language is a complex and multifaceted construct. It typically includes the production of speech sounds and patterns (phonetics and phonology), the words and associated knowledge (semantics), and the systems for combining parts of words together and words into sentences to convey meaning (grammar; Bates et al., 1992; Hoff, 2013). Research examining these underlying components of language has uncovered diverse developmental trajectories starting in early childhood (Huttenlocher et al., 2010). Indeed, children develop language relatively early compared to other domains, not only because it necessary for communication (Cates et al., 2012), but also because it is foundational for the acquisition of higher-order skills (Kuhn et al., 2014). The first 5 years constitutes a sensitive period of development, as children increase their language learning at a rapid rate due to increased neural plasticity in the brain (Knudsen, 2004). By age four, language development becomes more stable and is highly predictive of language abilities in adolescence (Bornstein et al., 2014). For instance, one study demonstrated that children’s oral narrative skills measured at school entry were related to their reading comprehension performance at age 16 (Suggate et al., 2018). Early language skills are also linked to a host of outcomes across developmental domains. These include cognitive skills (e.g., executive functioning), academic achievement, and socioemotional competence (Bleses et al., 2016; Hentges et al., 2021; Bruce and Bell, 2022). Thus, it is important to provide experiences that promote language acquisition early in life to ensure children are set on a path for success.

Children’s language abilities are influenced by both genetic and environmental factors (Hoff-Ginsberg, 1998; Hayiou-Thomas et al., 2012). Notably, the amount of speech children hear has a direct impact on their developing language, suggesting their language development is shaped by social contexts (Mahr and Edwards, 2018). To illustrate, Hart and Risley (1995) documented a 30-million-word gap between children from families with higher socioeconomic status (SES) and children from families with lower SES. Moreover, neurological studies indicate children who experience more conversational turns with adults have more complex speech, which is reflected in brain functioning that underlies language processing (Hutton et al., 2017; Romeo et al., 2018). A large body of observational work has also examined specific aspects of adult interactions that affect language development (see Golinkoff et al., 2019 for a review). Recently, a meta-analysis revealed moderate effects of the quality (e.g., diversity and reciprocity) and quantity (e.g., number of words) of language inputs in the home environment on children’s language development (Anderson et al., 2021). Specifically, conversational interactions between infants and adults account for significant variation in vocabulary in school-age children (Gilkerson et al., 2018). Yet, children are exposed to many sources of language input that are not captured by parent–child interactions, potentially underestimating the language environments of children from low SES backgrounds (Sperry et al., 2019). For instance, “social determinants of language development” outside of the immediate family include community resources, educational programs, and public policies (Di Sante and Potvin, 2022). Notably, the majority of children who are under the age of five spend their day in some type of non-parental care (Pilarz, 2018). Therefore, it is necessary to consider the features of child care and education settings that impact language acquisition.

Classroom and home environments provide very different language opportunities for children. Comparisons between child care and home language environments generally reveal more language interactions occurring between children and caregivers in the home relative to teachers and children in the classroom (Larson et al., 2020). Still, research has documented an association between language exposure in early childhood education settings and children’s language abilities and growth (e.g., Turnbull et al., 2009). For example, one study projected a difference of 100 adult words heard per 5 min (equivalent to approximately 1,800,000 words per year) that could be attributed to variation in prekindergarten classroom language environments (Duncan et al., 2023). Children from families with low SES are more likely to experience language of more limited complexity and diversity in the home and school context (Neuman et al., 2018; Duncan et al., 2023). This may be, in part, due to families having limited access to high-quality early childhood education in higher poverty communities (Bassok and Galdo, 2016). Targeting the quality of early learning environments in areas with greater socioeconomic disadvantage, and dedicating more public funding to these communities, may help to reduce the observed income quality gradient in ECE (Hatfield et al., 2015; Cloney et al., 2016). For example, research indicates that improving community-level social determinants, such as increasing the supply of child care, may elevate children’s literacy scores (Lipscomb et al., 2019). Thus, ensuring children have access to rich language environments inside and outside of the home may be one promising approach to enhancing equity in language development.

## Assessments of children’s language environments

Assessments of early childhood learning environments serve multiple purposes—they describe the quality of language environments, help to identify best practices, evaluate programs and interventions, and may even guide public policy (Lambert et al., 2006; Halle et al., 2010). Yet, there is ongoing debate as to which components of educational environments are most essential to assess and meaningfully link to children’s short- and long-term outcomes (Layzer and Goodson, 2006; Burchinal, 2018). Historically, measures have been classified based on whether they assess structural characteristics of classrooms (e.g., adult-child ratios) or the process of teaching (e.g., teacher-child interactions; Mashburn et al., 2008). The most frequently administered assessments of children’s learning environments are observational in nature and typically offer a one-time general snapshot of both the physical space and interactions within the setting (see Halle et al., 2010 for a review). For example, the Early Childhood Environment Rating Scale–Revised (ECERS–R) was designed as a global measure of classroom quality that assesses both structural and process aspects of the early childhood environment, including language reasoning (Cryer et al., 2003). However, the ECERS–R only weakly correlates with children’s language outcomes (*r* = 0.05; Brunsek et al., 2017), providing little evidence that using the ECERS – R for quality improvement purposes will result in the intended effects among children. Researchers have discovered that domain-specific measures focused on the quality of instruction and stimulation in specific content areas, like language and literacy, are more promising for guiding practice (Burchinal et al., 2021). Even so, domain-specific measures of language environments remain less commonly assimilated into accountability systems than global assessments of quality (Mitchell, 2016).

The early language environment is comprised of classroom features (e.g., activity settings), characteristics of teachers (e.g., pedagogical orientations), and interactions that support language development (e.g., input, responsivity, feedback; Smith and Dickinson, 1994; Wiggins et al., 2007). Research indicates that teacher communication-facilitating behaviors, those that create and sustain engagement in conversational turns, are the most powerful predictor of growth in children’s vocabulary from preschool to kindergarten (Cabell et al., 2015; Justice et al., 2018). Indeed, teachers can bolster children’s language development by providing opportunities for children to speak and extending their responses (Huttenlocher et al., 2002). Given that children ages 4–6 spend approximately 20–30% of their day learning in language and literacy domains, assessments that capture overall exposure to language and support for rich conversations are most promising for examining the features of environments that scaffold language development (Pelatti et al., 2014). We recognize that other assessments have been developed to include an item, subscale, or dimension for measuring language environments [e.g., the Classroom Assessment Scoring System (CLASS); Pianta et al., 2008, Teacher Behavior Rating Scale (TBRS); Landry et al., 2000] or features of teacher language input [e.g., Code for Interactive Recording of Children’s Learning Environments (CIRCLE); Atwater et al., 2009]. However, for the purpose of keeping our review brief and focused on the topic at hand, we have decided to only highlight domain-specific assessments of language/literacy. Therefore, we describe characteristics of frequently utilized domain-specific assessments of early childhood language environments in terms of their measurement properties. Importantly, we make the distinction between classroom-level assessments that provide information about the quality of instructional strategies teachers employ to engage all children in literacy activities, and child-level assessments that evaluate the quality and quantity of linguistic interactions individual children have with their teachers and peers.

### Classroom-level assessments

Classroom-level assessments of early language environments measure the widespread opportunities that are available to support children’s language development. These assessments typically include multiple dimensions of the early language environment, including the context, materials and activities, and instructional practices (see Table 1). Given their focus on general routines and exercises, these assessments offer a macro view of the overall educational processes in classrooms and are predominantly observation based. Because of the risk of rater bias, administrators of classroom-level assessments may need a certain level of education, experience, and familiarity with early childhood development and learning, and must complete training on implementation procedures. However, the length of observation period that is necessary for obtaining reliable estimates can vary for classroom-level assessments. For example, the Early Language and Literacy Classroom Observation Pre-K assessment (ELLCO) assesses the degree to which children receive optimal support in language and literacy development (Smith et al., 2008). Administrators need a strong background and understanding of children’s language and literacy development, classroom teaching experience, and must complete 9 h of training. Yet, the observation only takes approximately 1.0–1.5 h to administer. Alternatively, the Classroom Language Environment Observational Scale (CLEO) captures both implicit language supports and explicit language instruction (Phillips et al., 2018). Administrators should hold a bachelor’s degree or higher and have experience teaching or observing early childhood classrooms. Training includes one full day of instruction plus 6–8 weeks of practice to establish reliability, and the CLEO observation lasts throughout the entire classroom day. Despite these slight variations, both the ELLCO and CLEO have demonstrated moderate to strong internal consistency (Smith et al., 2008; Phillips et al., 2018).

**Table 1:** Features of commonly utilized language environment assessments.

			Domains					Administration		
Type	Assessment	Authors	Setting	Materials	Activities	Instruction	Interactions	Method	Training	Length
Classroom-level	OMLIT	Goodson et al. (2006)	✓	✓	✓	✓		O	4–8 h/subscale	At least 3 h
	ELLCO	Smith et al. (2008)	✓	✓	✓	✓		O + I	9 h	1.0–1.5 h
	CLEO	Phillips et al. (2018)		✓	✓	✓		O	Full day +6–8 weeks study	Full day
	E-LOT	Grehan and Smith (2004)	✓	✓		✓		O	Unknown	90 min
	SELA	Smith et al. (2001)		✓	✓	✓	✓	O + I	Several hours	3.0–3.5 h
	CASEBA	Freedson et al. (2011)		✓	✓	✓	✓	O + I	1 day +2 days coding	3–4 h
	ELLECCT	Weadman et al. (2021)				✓	✓	O	Unknown	< 20 min*
Child-level	ISI	Connor et al. (2009)	✓		✓	✓	✓	OR	Half day	6–12 min/child
	LISn	Atkins-Burnett et al. (2010)	✓			✓	✓	O	2 days +4 h study	5 min/child
	LENA	Xu et al. (2009)					✓	OR	Unknown	Full day/child

Setting, context or orientation (e.g., whole group, child initiated); Materials, opportunities (e.g., book area, visible print); activities, behaviors (e.g., book reading); instruction, facilitation strategies (e.g., open-ended questions, directives); interactions, verbal communications (e.g., quality or quantity); O, observation; I, interview; OR, observational recording; table adapted from Halle et al. (2010); unknown, information could not be found; *time of shared book reading.

### Child-level assessments

Child-level assessments of early language environments measure the language experiences that individual children are exposed to. These assessments typically focus on the quality and quantity of language interactions and may also capture the context of instruction or activities (see Table 1). Given their focus on relational components, these assessments offer a micro view of the child and their immediate exchanges with peers and adults. Because they predominantly rely on technological applications for observations, administrators of child-level assessments need less direct classroom experience or child development knowledge but may be required to complete training to master the software and coding procedures. There is less risk for rater bias, so reliable estimates can be obtained with shorter lengths of observation periods. For example, the Individualizing Student Instruction Classroom Observation System (ISI) is a video observation and coding system that assesses foundational and instructional elements of the language and literacy classroom environment (Connor et al., 2009). The ISI requires one half day workshop to introduce the assessment and software, but it is most successful with ongoing coaching and professional development (Connor and Morrison, 2016). Video observations on the ISI should record about 6–12 min per target child. Alternatively, the Language Environment Analysis System (LENA) is an audio recording device that quantifies the number of words heard, child vocalizations made, and conversational turn taking (Xu et al., 2009). A basic training on how to use the device and audio processing software is required to purchase the LENA technology. Observations should be a minimum of 10 min long, but the LENA device will record up to 16 h. Both the ISI and LENA have demonstrated moderate to strong internal consistency (Connor et al., 2009; Xu et al., 2009).

## Research gaps and opportunities

Despite the wide range of assessment options, ongoing debates regarding the merit of observational methods (e.g., Purpura, 2019) have contributed to a lack of consensus regarding which aspects of language environments are most promising to assess. Since the formative Hart and Risley study (1995), total amount of language heard remains of interest to researchers (e.g., Sperry et al., 2019; Duncan et al., 2023). Indeed, frequent input of directed speech is important for building early vocabulary and language-learning processes (Mahr and Edwards, 2018). However, there is also evidence indicating the quality of talk is of greater importance for children’s language development than purely the number of words heard (Golinkoff et al., 2019; Anderson et al., 2021). Features of adult speech, such as lexical diversity and reciprocity, enable children to adopt word-learning mechanisms (Hirsh-Pasek et al., 2015; Newman et al., 2016). While the total amount of talk may be easier to code with language processing devices (e.g., the LENA), the quality of talk often requires more intensive researcher coding or subjective ratings of the experiences (e.g., Justice et al., 2018; Anderson et al., 2021). Possibly in between the pure counts of adult words heard and quality of language interactions would be the number of conversational turns children experience. These are typically defined as the instances of back and forth between the child and an adult. Conversational turns—usually conceptualized as quantitative metrics—have been linked to number of key child outcomes and brain development (Gilkerson et al., 2018; Romeo et al., 2018), and have been shown to have larger effect sizes than pure numbers of adult words heard (Duncan et al., 2023). Still, more research is needed that compares approaches to determine which provides the greatest return on investment both in terms of predictive power and information gained relative to effort expended.

Widespread adoption of language environment assessments has also, in part, been hindered by limited direct application in program evaluation research. As previously noted, interventions that focus on conversational interactions elicited by adults have the strongest potential to foster vocabulary development (Dowdall et al., 2020). For example, dialogic reading has been found to encourage the use of a larger vocabulary, greater conceptual discussion, and longer sentences, which in turn, can impact the neural networks underlying language development (Wasik et al., 2006; Hadley et al., 2022). Yet, evidence suggests that Head Start teachers rarely ask open-ended prompts or wait for responses during shared book reading (Hindman et al., 2019). Language environment assessments can therefore be leveraged for the purpose of documenting existing processes and developing strategies for improving interactions. For instance, preliminary results indicate the LENA Start Program—designed to improve knowledge about the importance of the early language environment and provide tips for enriching the early language environment—can effectively increase the number of conversational turns between children and their parents through enhancing parental beliefs about the quality and quantity of language input (Cunha et al., 2023). Therefore, researchers should evaluate whether strategically integrating language environment assessments into large-scale interventions increases awareness and positive attitudes about the importance of conversational turns and impacts children’s learning outcomes.

Relatedly, researchers can utilize language environment assessments to examine the ecological validity of language interventions and programs designed to improve the quality of early learning. For example, it is unclear whether effects of interventions delivered by trained research assistants transfer when administered by practitioners in real world situations (Piasta et al., 2020a,b). Indeed, a systematic review synthesizing results from early language interventions implemented by caregivers and parents of young children revealed that only half of studies observationally assessed changes in the quality of the language environment (Greenwood et al., 2020). However, it is critical that language environment assessments are validated for this purpose and can detect discrete changes in all critical aspects of language environments that are known to predict language development, such as diversity and complexity of vocabulary, conversational turn-taking, verbal responsivity, and the supports for primary and secondary language learning (Anderson et al., 2021). Thus, it is important to continue to develop and refine language environment assessments before administering them to examine the fidelity of implementing interventions or programs within authentic settings. Both efforts will provide evidence of the suitability of existing measures to detect targeted changes in language environments.

The effects of language interventions on child- and classroom-level processes have also been under-evaluated in the literature. Previous work indicates the classroom context is an important factor that influences the frequency of language stimulation practices (Turnbull et al., 2009). For instance, in a meta-analysis of language interventions delivered to children between 4 and 9 years old, Rogde et al. (2019) found that studies with small-group interventions demonstrated larger effects on oral language skills than whole-classroom interventions or those involving larger groups. These results imply that high-quality interventions delivered to small groups can be beneficial for children’s language development. However, it is possible for more targeted language interventions to have spillover effects that enhance the larger classroom language environment, or impact future cohorts of students in the classroom (Cilliers et al., 2022). Peers may also make significant contributions to one another’s language skills by serving as language models (Perry et al., 2018; Washington-Nortey et al., 2022). Therefore, utilizing both child- and classroom-level language environments assessments to understand the potential for educational interventions to have a ripple or cascading effect may lead to significant discoveries regarding persistence and fadeout.

Language environment assessments are also somewhat limited in their ability to capture the breadth of classroom practices and strategies that promote language development. Beyond interactive or dialogic book reading, approaches involving speech and language therapists have been shown to be effective interventions for supporting language development (Dobinson and Dockrell, 2021). Moreover, a meta-analysis revealed that language and literacy focused professional development in preschool had a medium to small effect on process quality and children’s phonological awareness (Markussen-Brown et al., 2017). However, many language environment assessments are not intended to capture the processes targeted by professional development (Justice et al., 2018), or the direct impacts of coaching or instruction provided by other specialized professionals. Therefore, a complementary line of work should center on the development and modification of language environment assessments to align with a broader conceptualization of what constitutes language supports.

Finally, there are critical gaps concerning the applicability of language environment assessments within diverse educational settings, including classrooms that serve dual language learners (DLLs), that must be filled (White et al., 2020). For example, one analysis determined that most studies on the LENA system have been conducted with English-speaking children (Ganek and Eriks-Brophy, 2018). A lack of representation is also apparent across language intervention research. In a systematic literature review, Walker et al. (2020) demonstrated that less than a quarter of language intervention studies in early education programs reported information about the race or ethnicity of children participating in the intervention, and only a quarter of samples included children who were DLLs. Further, they discovered that only 12% of studies reported on the racial/ethnic backgrounds of the early childhood personnel administering the interventions. Given that DLLs acquire their first and second language best when delivered instruction in their home language (e.g., Barnett et al., 2007; Partika et al., 2021), it is important that language environment assessments capture the instructional practices that facilitate first and second language development, such as the use of contextualized language (Sawyer et al., 2018). Thus, research that addresses these shortcomings may also help to clarify how to appropriately incorporate language environment assessments into mainstream education.

## Considerations for practice

In addition to the limitations of current research on early childhood language environments, there are practical constraints that may be impeding widespread uptake of assessments. Teachers may lack a fundamental understanding of what constitutes oral language and emergent literacy (Weadman et al., 2023), as well as the skills to evaluate their own assessment practices and professional development needs (Hill, 2017). These competencies are critical to develop because educator language and literacy knowledge has been shown to be correlated with more desirable classroom practices and children’s language outcomes (Piasta et al., 2020a,b). Researchers argue there is a need for consistent training on language and literacy content and the benefits of teacher talk for children’s language development in educator preparation programs (Weadman et al., 2021). Moreover, ongoing professional development should focus on effective language facilitation and conversation strategies that preschool teachers can implement and should offer practice connecting this procedural knowledge to “in the moment” situations (Mathers, 2021). For example, one study used the Emergent Literacy and Language Early Childhood Checklist for Teachers (ELLECCT) assessment to demonstrate how teachers often miss opportunities to target children’s phonological awareness during shared book reading (Weadman et al., 2022). Therefore, early childhood language environment assessments may serve as a useful framework for training and professional development to help inform teachers of their use of practices associated with language development (Franco et al., 2019).

Choosing an assessment that aligns with program goals and meets the needs of children and educators can, however, be a challenge in and of itself (Zaslow et al., 2009). For instance, the ELLCO provides teachers with an understanding of the quality of the language and literacy environment of the classroom, while the LISn offers details on the quality of interactions between peers and adults, including the frequency and types of languages spoken. There are also tradeoffs according to the amount of information ascertained with reasonable effort. Classroom-level metrics can be captured by only observing and rating the teacher (i.e., the behaviors the teacher shows that would impact all children in the classroom). Conversely, child-level metrics would potentially better capture the unique experiences of children within the classroom, as opposed to assuming all children have the same experiences. Depending on whether the purpose of the assessment is to understand granular language engagement processes or general indices of the quality of language environment, some measures may be more sensitive to pick up discrete characteristics than others (Justice et al., 2018). Thus, one consideration may be whether to administer classroom-level assessments to inform universal teaching practices (Baker and Páez, 2018), or child-level assessments to provide guidance on individualized interventions for children (Franco et al., 2019).

Additionally, it may be unrealistic for educational programs to accumulate the capital necessary for precise measurement and communication of language environment assessment findings. As described earlier, many assessments require extensive training and experience within early childhood classrooms, and programs may not have staff with relevant background to dedicate to this purpose (Ackerman, 2019). For instance, the protocols for several assessments recommend administration by individuals with certain education levels or observation history. It may not be feasible for a teacher to administer individual assessments when they must also manage the classroom environment and facilitate children’s learning and development. Similarly, substantial training is often needed to become a reliable observer and maintain adherence to the procedures of language environment assessments (Zaslow et al., 2009). Meeting these prerequisites can be costly and labor intensive, especially for programs that do not have a robust administrative infrastructure in place. The amount of support a program receives from their leadership to overcome these obstacles may be a critical factor in determining assessment participation (Derrick-Mills et al., 2014).

Even after a language environment assessment has been chosen and implemented, programs may need specific resources to leverage the data to inform professional development. At a very basic level, human capacity is needed to analyze and translate data, which sometimes involves individuals who have pertinent technical skills to interpret statistics and graphs (Isaacs et al., 2015). Programs may also need staff who can manage complex equipment, technology, and datasets. For example, child-level assessments like the LENA and ISI rely on hardware for collecting data and software for analyzing and/or storing data and developing reports (Derrick-Mills et al., 2014). Beyond these logistical parameters, another challenge is effectively using the information to promote positive practices. One issue that can arise is teachers may assume they should emphasize only the discrete aspects of instruction addressed through the assessment itself rather than supporting broader integration (Hirsh-Pasek et al., 2005). Thus, hiring coaches who have relevant funds of knowledge to make developmentally appropriate and evidence-based recommendations may be essential (Ackerman, 2019). As Mathers (2022) contests, adoption of an observation tool itself cannot guarantee successful application without the focus on professional learning.

Another barrier that may be necessary to overcome is creating buy-in around language environment assessment use. For example, research suggests that teachers generally do not find kindergarten readiness assessments beneficial for instruction, and further, they view them as administratively burdensome and as taking time away from instruction, rather than contributing useful information for practice (Schachter et al., 2020). Although there has been limited investigation into teacher perspectives on classroom environment assessments, one study found that administrators experience difficulties finding the time to conduct observations of classroom quality, but they report using the data for a variety of purposes, including individual teacher development (Zweig et al., 2015). One framework that has been proposed for conceptualizing emergent literacy data practices is through examining: (1) how teachers prefer to gather data, (2) how they interpret data, (3) and how they use the data (Schachter and Piasta, 2022). Given that educators play a critical role in supporting young children’s language development, it will be important for administrators to incentivize the implementation of language environment assessments and be prepared to engage in best practices around collecting and using data.

A final consideration for educators selecting assessments is whether to operationalize high-quality language environments beyond simply examining average levels of exposure. Studies have documented substantial variation in language environments within and across school days (Chaparro-Moreno et al., 2019; Duncan et al., 2020), which may have implications for vocabulary development. For instance, research suggests that inconsistency in instructional quality, including concept development, language modeling, and quality of feedback, may be negatively associated with children’s vocabulary development in prekindergarten (Finders et al., 2021). In early education settings where teachers do the majority of talking, persistent language use by the teacher could reflect a lack of responsivity. Indeed, one study demonstrated that children spend very little time in the preschool classroom engaged in responsive interactions with their teachers (Dickinson et al., 2008). Responsiveness, which refers to promoting reciprocity in language interactions, involves teachers providing children opportunities to use language by asking open-ended questions and modeling talk, and is one of the strongest indicators of DLL’s language development (Kane et al., 2023). Therefore, the degree to which assessments are designed to pick up on small changes in the quality of language provision throughout the day may be important factor to weigh when determining which measure to utilize. To sum, there are many factors that need to be balanced when selecting a language environment assessment, and without adequate resources and support, programs may struggle to navigate this complex territory.

## Conclusion

Despite a robust literature underscoring aspects of language input that matter for children’s language development, language environment assessments have not been widely integrated into early childhood programming. In this mini review, we argue that in order to promote high-quality learning experiences within early education settings, we must be able to operationalize the essential features of these environments through assessment. Therefore, we provide concrete considerations for research and practice that have the potential to build awareness of and capacity to effectively utilize language environment assessments to improve the quality of language environments for diverse learners. We maintain that incorporating assessment practices into early education is a critical first step toward addressing socioeconomic disparities in language development and enhancing equitable opportunities for children worldwide (Jemeli and Fakandu, 2019).

## Author contributions

JF, EW, and RD contributed to conception of the manuscript and wrote sections of the manuscript. JF developed the framework and overall structure. All authors contributed to manuscript revision, read, and approved the submitted version.

## Funding

Publication of this article was funded in part by Purdue University Libraries Open Access Publishing Fund.

## Conflict of interest

The authors declare that the research was conducted in the absence of any commercial or financial relationships that could be construed as a potential conflict of interest.

## Publisher’s note

All claims expressed in this article are solely those of the authors and do not necessarily represent those of their affiliated organizations, or those of the publisher, the editors and the reviewers. Any product that may be evaluated in this article, or claim that may be made by its manufacturer, is not guaranteed or endorsed by the publisher.
